# Multivariate Chemometric Comparison of Forced Degradation and Electrochemical Oxidation LC–MS Profiles of Maraviroc

**DOI:** 10.3390/molecules28031195

**Published:** 2023-01-25

**Authors:** Michał Wroński, Jakub Trawiński, Łukasz Komsta, Robert Skibiński

**Affiliations:** Department of Medicinal Chemistry, Faculty of Pharmacy, Medical University of Lublin, 20-090 Lublin, Poland

**Keywords:** chemometrics, Q-TOF, mass spectrometry, UHPLC, photodegradation, degradation studies

## Abstract

In this study, nine forced degradation products of maraviroc were found using chemometric analysis. This antiretroviral drug was subjected to photolytic, oxidative, as well as neutral, basic and acidic hydrolysis stress conditions. Additionally, its electrochemical transformation on platinum, gold and glassy carbon screen-printed electrodes was examined. This study showed that maraviroc is especially susceptible to UVA, H_2_O_2_ and electrochemical degradation, while being resistant to neutral and acidic hydrolysis. A cluster analysis showed that the electrochemical transformation, with particular reference to the platinum electrode, is able to partially simulate the forced degradation processes, especially in the context of redox reactions. These findings indicate that the electrochemical methods can be considered as quick and relatively low-cost supplements to the commonly applied forced degradation procedures.

## 1. Introduction

Under various stress conditions, pharmaceutical compounds can be converted to potentially toxic derivatives, and those formed during the manufacturing or storage stages pose a risk to the safety of therapy because of a risk of adverse reactions in patients. Impurities in bulk and dosage forms should be taken into account when assessing the toxicity of a formulation, as well as during investigation of the pharmacological-toxicological profile of the active substance [1]. Forced degradation tests are designed to indicate a molecule’s potential to decompose in stress conditions, such as light, humidity, temperature, different pH, some reaction with excipients or on contact with primary packaging [2]. The most common pathways of degradation are hydrolysis and oxidation caused by the ubiquitous presence of moisture and oxygen, so the degradation cannot be completely avoided in general [3]. The purpose of these studies is to predict drug shelf life and to recommend appropriate techniques for manufacturing and storing the drug, preventing a loss of effectiveness and the formation of toxic derivatives.

Chemometric analysis has a valuable place in drug stability studies [4,5,6,7]. Multivariate methods in analytical chemistry enable data reduction, grouping and classification of observations, as well as modeling of relationships that may exist between variables [8]. The application of chemometric tools such as Principal Component Analysis (PCA) or hierarchical cluster analysis (HCA) has proven to be efficient in obtaining better visualization of data even from low resolution and high noise levels of analytical measurements [9].

Direct electrochemical oxidation combined with mass spectrometry confirmed the potential to mimic certain forced degradation studies [10]. In direct electrochemical reactions, charged particles cross the interface between an electrode and a solution. The electrode can behave as a reducer or oxidizer, depending on the controlled potential. Electron transfer results in the oxidation or reduction of a substance when a positive or negative potential is applied, respectively. Electroanalysis coupled with screen-printed electrodes (SPE) is a technique that has become increasingly popular in recent years for on-site electroanalysis [11]. SPEs enable easily obtaining small-sized flat electrochemical cells typically consisting of three electrodes (working, reference and counter electrode) which are printed on a rigid material. These low-cost and versatile analytical instruments provide quantitative information quickly and in an easy manner, with high sensitivity and reproducibility [11,12].

Maraviroc is approved for the treatment of HIV infection by blocking the C-C chemokine receptor 5 (CCR5). This receptor is the main coreceptor by which the HIV-1 enters target cells [13]. Maraviroc is always used in combination with other antiretroviral agents and its use should be limited only to patients whose HIV strains use the CCR5 coreceptor or both the CCR5 and CXCR4 coreceptors.

So far, only in a few studies has the behavior of maraviroc under stress conditions been tested, but with no attempt to identify the products of degradation reactions.

Chakravarthy et al. [14] concluded that maraviroc is sensitive to oxidative conditions and degraded significantly when exposed to an acid medium, while remaining resistant to moisture, heating, alkaline medium and UV–VIS radiation. In similar work performed by Sait et al. [15], maraviroc was found to be most sensitive to alkaline hydrolysis (3 unknown degradation products were observed), it slightly degraded after incubation in neutral, acidic and oxidative media, while being stable in moisture and under UV–VIS radiation. In Chilukuri et al. [16], maraviroc was incubated in neutral, acidic, basic and oxidative conditions, and its behavior under moisture and UV–VIS radiation was also verified. Maraviroc was found to be sensitive to acid hydrolysis, forming a product that was identified as a known impurity based on retention time. In an oxidizing environment, one product was formed, identified as N-oxide. Although these studies demonstrated the stability of maraviroc under UV–VIS radiation, in our previous work [17], we investigated the stability of maraviroc in ultrapure water and river water treated with these conditions, identifying 12 and 14 resulting degradation products, respectively.

Taking this into account, it seems to be necessary to evaluate the behavior of maraviroc under various stress conditions, as well as to elucidate the structures of the formed transformation products. In addition, optimization of electrochemical parameters to mimic responses under stress conditions was carried out by applying platinum, gold and glassy carbon SPE. The multivariate chemometric analyses (PCA and HCA) of the forced degradation profiles of maraviroc enabled identification of new degradation products of this drug as well as choice of the optimal electrochemical experiment.

## 2. Results and Discussion

### 2.1. Optimization of the LC–ESI–MS/MS and EC Methods

Based on previous studies, the chromatographic conditions were optimized to make the analysis time relatively short and to obtain a good separation of the obtained products [17]. Finally, aqueous formic acid (0.1%) and acetonitrile were used as eluting solvents. Separation was achieved by means of a gradient profile described in Section 3.4.

Preliminary optimization of the EC method on SPE electrodes was achieved by performing electrochemical experiments at different potentials, using different pH of electrolytes and acetonitrile content in a water medium. Finally, experiments were performed using a solution of phosphate buffer at pH 7.4 with acetonitrile 50:50 (*v*/*v*) and a potential of 1400 mV was applied.

### 2.2. Chemometric Study

**Figure 1:**
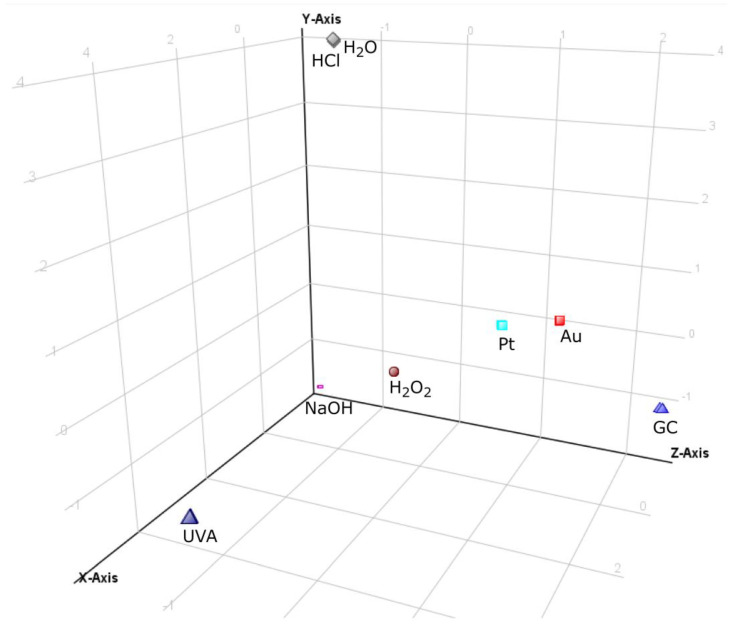
The 3D PCA plot of forced degradation profiles of maraviroc in tested conditions.

All the obtained chromatographic profiles (40 chromatograms: 8 conditions in 5 replicates) registered in the TOF (MS) mode were the input into MPP software in order to carry out multivariate chemometric analysis. Analysis resulted in 103 specific ions (entities). After applying built-in MPP filtration, which included filtering by flags, sample abundance, followed by setting the fold change (FC) threshold on the level not less than 2.0, 11 entities were finally selected for the chemometric study. The PCA analysis of these data showed a visible categorization of all the analyzed groups of the forced degradation samples (Figure 1) apart from neutral (H_2_O) and acidic (HCl) stressed samples which were very close to each other. These two samples were outliers because under these conditions, maraviroc proved to be completely stable, not leading to the formation of any transformation products (TPs). Electrochemical test samples were clustered relatively closely together and were situated not far from the oxidized (H_2_O_2_) and basic (NaOH) stressed samples (although samples obtained on GC SPE were noticeably most distant from any of the forced degradation samples). On the other hand, electrochemical test samples were separated from the UVA samples, which have departed from the other samples because of the largest number of products. In addition, due to the high stability of maraviroc in acid and neutral stress conditions, these samples also separated from the rest of them. PCA analysis clearly shows that the electrochemical test sample on the platinum and gold electrodes were closer to the forced degradation test samples than samples from GC experiment. In the presented PCA analysis, the first three components (PC) explained 86.22% of the total variance (PC1—43.25%; PC2—26.43%; PC3—16.54%).

**Figure 2:**
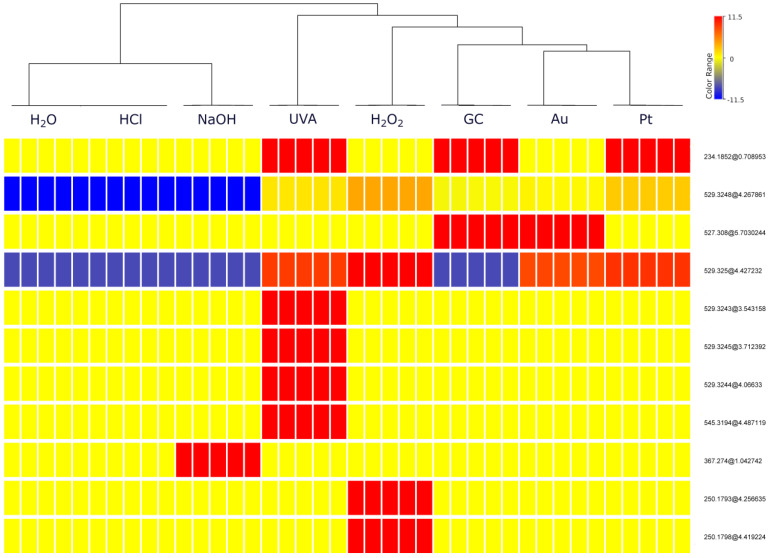
Hierarchical cluster analysis (HCA), (entities abbr.—mass@retention times). Each rectangle represents one entity (i.e., a transformation product or its fragment), and a color of bar—normalized concentration, ranging from very low to moderate and very high, which are shown in blue, yellow and red, respectively.

To further visualize whether electrochemical reactions mimic degradation processes, the dataset was subjected to hierarchical cluster analysis (HCA). HCA was conducted using Ward’s linkage with the Euclidean distance. Based on the HCA results (Figure 2), out of 11 entities, 9 were selected (Table 1) and identified through further analysis using Q-TOF MS. Two excluded entities with a mass of 250.1793 Da at a time retention of 4.26 min (251.1867 *m*/*z*) and 250.1798 Da at a time retention of 4.42 min (251.1870 *m*/*z*) turned out to be ions derived from two other products—529.3248 and 529.3250 Da, respectively. The dendrogram produced from HCA showed separation on two main groups. Neutral, acid and basic stress samples in which minor changes have been observed are clustered together. EC samples are clustered together with oxidative and UVA stress conditions. It is noteworthy that the most transformation products were observed in these samples, indicating that EC methods can be a useful approach for mimicking forced degradation reactions. The heatmap shown in Figure 2 illustrates the differences between these clusters. Warmer colors (red) correspond to higher TPs abundance values and colder colors (blue) indicate lower values. Those shown in the heatmap legend (−11.5, 0, 11.5) represent the natural logarithm values of the abundance. The neutral and acidic stress samples contained the lowest abundances of most of TPs. The most similar to them are basic stress samples, which were distinguished by the presence of a high concentration of one TP. In contrast, the largest abundances of TPs are found in the UVA samples and H_2_O_2_ samples were close to them. On the other hand, all electrochemical samples, despite differences between abundances of specific products, were quite similar to each other. Among these samples, those obtained on the Pt SPE proved to be the closest to the forced degradation samples, in particular to H_2_O_2_ stress samples.

### 2.3. Identification of Forced Degradation Products

In order to identify the degradation products obtained during the experiments, MS analysis was generally carried out using the auto MS/MS mode with positive-mode electrospray ionization. During all forced degradation and electrochemical experiments, nine products were detected and further selected for chemometric analysis. In addition, different amounts of collision-induced dissociation (CID) energy were used to obtain the most diverse TP fragmentation spectra. As shown in Table 1, the accurate masses of selected TPs did not exceed an acceptable error limit equal to 7.0 ppm. Seven of the products identified in this study are previously described hydroxylation or dealkylation results of photochemical transformation. However, two of them (TP2 and TP9) were not reported so far. As we noted in our previous study, maraviroc and most of its TPs are prone to form multiply charged ions in ESI source because of the presence of many nucleophilic atoms [17]. In some cases fragmentation of the double-charged ions provided more information about the structure than the single-charged ones, so it was decided to use sometimes the fragmentation data of double-charged ions to study the degradation profiles of maraviroc in a better way. The MS/MS spectra and the proposed structures of the detected TPs are shown in the Supplementary Material.

The fragmentation path of the maraviroc molecule (Figures S1 and S2), also often observed in the spectra of its TPs, begins with the easy elimination of the alkyltriazole (ATZ) molecule (389.2397 *m*/*z*), which is followed by the removal of azabicyclooctane (ABO) (280.1512 *m*/*z*) and one fluorine atom (260.1438 *m*/*z*). Further fragmentation after difluorocyclohexyl (DFCHX) amide disconnection leads to the formation of ions at 117.0703, 106.0656 and 91.0540 *m*/*z* derived from the 1-phenylpropanoamine system. Fragmentation from the opposite side is represented by the ions at 185.1270 and 226.1575 *m*/*z*, which correspond to the disconnection of DFCHX carbonyl and DFCHX amide, together with the ATZ moiety, respectively. Fragments from the decay of the ATZ-ABO moiety (205.1428, 124.1121 and 79.0554 *m*/*z*) are also visible in the MS/MS spectrum (Figures S1–S17).

TP1 is the product of dealkylation, still containing ABO and ATZ moieties [17]. Its MS/MS spectrum (Figure S3) contains an ATZ fragment (126.0965 *m*/*z*) as well as fragments derived from ABO fragmentation (67.0566, 82.0651 and 93.0697 *m*/*z*).

TP2 is an amine that has not been described previously, formed by the alkaline hydrolysis of an amide bond. Its fragmentation begins with the loss of ATZ, resulting in formation at 243.1893 *m*/*z*. The main ion in its MS/MS spectrum (Figure S4) represents methylated ABO (124.1122 *m*/*z*). The other two ions visible in the MS/MS spectrum (96.0823 and 70.0672 *m*/*z*) originate from a partially degraded ABO fragment.

TP3, TP4 and TP5 are products of phenyl ring oxidation [17]. However, MS/MS spectra do not enable determining the exact location of their hydroxylation. Both MS/MS ‘single-charged’ (Figures S5, S7 and S9) and ‘double-charged’ spectra (Figures S6, S8 and S10) are quite similar. Direct evidence of hydroxylation of the phenyl ring is provided by the ion at 133.0620 *m*/*z* (133.0641 *m*/*z* in the case of TP4 and 133.0638 *m*/*z* in the case of TP5) and also by the ion at 122.0589 *m*/*z* in the case of TP4. Different ions presenting gradual fragmentation of unchanged ABO, ATZ and DFCHX moieties indirectly confirm that hydroxylation did not take place within them 

In the case of TP6 and TP7, the hydroxylation took place in the ABO moiety [17]. Their single-charged (Figures S11 and S13) and double-charged spectra (Figures S12 and S14) are quite similar and do not enable presenting the exact location of additional hydroxyl group. The hydroxylated ABO fragment is represented as the ion at 126.0948 *m*/*z* (126.0933 *m*/*z* in the case of TP7). Both ions at 251.1860 *m*/*z* (251.1867 *m*/*z* in the case of TP7), containing the hydroxylated ABO-ATZ moiety in combination with the ions at 106.0652, 117.0693, as well as 280.1512 *m*/*z* (106.0656, 117.0701 and 280.1511 *m*/*z* for TP7) containing the unchanged phenylalkyl and DFCHX amide moiety, support this information in both spectra.

Fragment 514.2988 in spectrum TP8 (Figure S15) corresponds to the parent compound, which indicates presence of two relatively unstable nitrogen-oxygen bonds that can be present with N-oxides [17]. Moreover, unoxidized fragments at 280.1550 and 389.2441 *m*/*z* exclude hydroxylation of ABO system. In the ‘double-charged’ MS/MS spectrum (Figure S16), double-charged ions at 140.0940 and 185.1193 *m*/*z* represent fragments with double and mono-oxidation in the ATZ moiety, respectively. Ions at 243.1861 *m*/*z* (not present in any other spectrum of hydroxylated derivatives) and 124.1092 *m*/*z* and fragments of DFCHX-amide (119.0640 and 144.0821 *m*/*z*) indirectly suggest oxidation in the ATZ moiety.

TP9 is the unique product formed only by electrooxidation, and it was not described so far. In its MS/MS spectrum (Figure S17), there are some unique ions indicating the presence of hydroxylation and dehydrogenation in the molecule. Fragments at 294.1297, 148.0765, and 131.0492 *m*/*z* represent the progressive elimination of the unchanged ABO-ATZ moiety, the DFCHX system and the amino group, respectively, demonstrating that changes have occurred in the propyl chain. The fragment at 131.0492 *m*/*z* indicates the only position where an oxygen atom could be attached to the propanoamine chain. The ion at 240.1364 *m*/*z* represents a fragment in which ABO-ATZ moiety detachment occurred along with deoxidation, confirming that oxidation took place in the propyl chain with the formation of an amide bond. Both the fragmentation ions of ABO (67.0554, 93.0703, and 110.0996 *m*/*z*), ATZ (126.1092 *m*/*z*), and DFCHX structures (99.0611 and 119.0966 *m*/*z*) indicate that these moieties have remained unchanged.

### 2.4. Degradation Pathway

**Figure 3:**
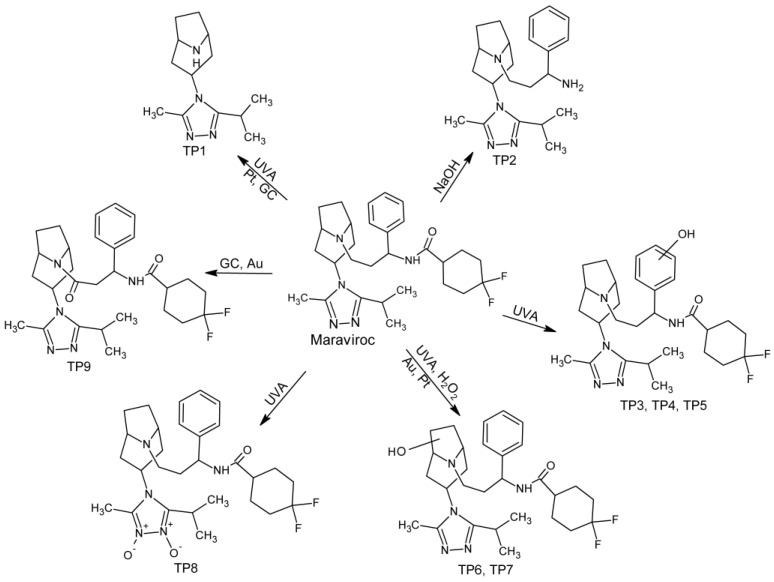
Forced degradation pathway of maraviroc in tested conditions.

During forced degradation studies, eight products were formed, and four of them were also produced by electrochemical reactions. One product, not previously reported in the literature, was formed only under electrochemical conditions. TP1 and TP2 are products of parent molecule fragmentation. Under the UVA irradiation, dealkylation has occurred leading to the formation of TP1. Its formation can also be achieved in electrochemical reactions on Pt and GC electrodes. In turn, TP2 is a newly discovered degradation product that was formed in an alkaline environment due to hydrolysis of an amide bond. TP3–TP7 were isomeric products of the addition of one oxygen atom to the parent molecule and TP8 is a double N-oxide derivate. All monohydroxylated and double N-oxide maraviroc derivatives were formed under UVA irradiation, but TP6 and TP7 were also formed under oxidative (H_2_O_2_) conditions. Alicyclic hydroxylated derivatives can be produced on Au and Pt electrodes. However, electrochemical methods did not succeed in producing aromatically hydroxylated derivatives, which are only formed under UVA radiation conditions. TP9 is the only product that is not formed under any of used stress conditions, but can be produced by electrochemical reactions on GC and Au electrodes. It is formed as a result of oxidation and dehydrogenation. The complete pathway of maraviroc degradation was presented in Figure 3.

## 3. Materials and Methods

### 3.1. Chemicals and Reagents

Maraviroc was purchased from MedChemExpress LLC (New York, NJ, USA). Acetonitrile for LC–MS, water for LC–MS and water for LC were purchased from Witko (Łodz, Poland). Hydrochloric acid and sodium hydroxide were purchased from POCh (Gliwice, Poland). The 30% hydrogen peroxide and sodium phosphate dibasic anhydrous salt were purchased from Sigma-Aldrich (St. Louis, MO, USA).

### 3.2. Forced Degradation Studies 

A stock solution of maraviroc dissolved in acetonitrile (2 mg mL^−1^) was diluted using proper solvent to finally obtain a working solution with a concentration of 10 µg mL^−1^.

All the hydrolytic and oxidative degradations were performed using 1 mL of working solution placed in a hermetically sealed 10 mL glass tube. Neutral (H_2_O), acidic (HCl) and alkaline (NaOH) stress conditions were subjected to shaking at 450 rpm using a thermoshaker at 80 °C for the entire 3 h of the experiment. Oxidative conditions in 1% hydrogen peroxide solution (*v*/*v*) (H_2_O_2_) were used for 3 h at room temperature. In a photodegradation study, 3 mL of working solution was placed in quartz capped cell (3.5 mL). Photolytic degradation was performed by irradiating the sample for 1 h with UVA using Dymax BlueWave^®®^ 200 UV Light-Curing Spot Lamp equipped with 200 W metal-halide bulb (Torrington, CT, USA), emitting 16.3 W cm^−2^ of UVA at the output, and irradiated from the distance of 10 cm. Pure solvents, without addition of maraviroc were also analyzed as the control samples. The applied conditions are summarized in Table 2.

### 3.3. Electrochemical Studies

An electrochemical analyzer Autolab/PGSTAT302N (Metrohm Autolab, Utrecht, Netherlands) controlled by the Nova 2.1. software was used for constant potential amperometry experiments. The electrochemical (EC) behavior of maraviroc was investigated on platinum (Pt), gold (Au) and glassy carbon (GC) SPE working electrodes (respectively 550, 220BT and 110; Metrohm DropSens) in which Ag-serves as a pseudo-reference electrode with platinum, gold and carbon electrodes as auxiliary electrodes, respectively. Measurements were carried out by applying a potential 1400 mV for 5 min, with a scan rate 100 mV s^−1^ and interval time 0.5 s. A solution of phosphate buffer at pH 7.4, and acetonitrile 50:50 (*v*/*v*) was used as a supporting electrolyte, in which a stock solution of maraviroc (2 mg mL^−1^) was diluted to prepare a working solution (0.01 mg mL^−1^). All the experiments were carried out at room temperature, by placing an 80 µL drop of solution on the surface of the SPE to cover all three electrodes.

### 3.4. LC–ESI–MS/MS Analysis

An Agilent high-resolution Q-TOF system series 6520 and UHPLC system series 1290 and Zorbax Eclipse-C18 HD (2.1 × 50 mm, dp = 1.8 μm) column was used for UHPLC–ESI-QTOF analysis. For control of the system, data acquisition, qualitative and quantitative analysis, MassHunter workstation software version B.06.00 (Agilent Technologies, Santa Clara, CA, USA) was used. To ensure accuracy in mass measurements, a reference mass correction was provided by using masses 121.050873 and 922.009798 as lock masses. The conditions for performing chromatography were optimized based on previous studies [17]. All of the chromatographic and spectrometric parameters are described in Table 3.

### 3.5. Data Preprocessing and Chemometric Studies

Five samples were recorded for each investigated stress condition of maraviroc. In order to clean up background ion noise in the data and extract the list of the characteristic ions of maraviroc degradation products, the molecular feature extraction (MFE) algorithm from Mass Hunter Qualitative Analysis software version B.06.00 (Agilent Technologies, Santa Clara, CA, USA) was used. After optimization of MFE parameters, the following settings were used: maximum 2 charge state of the analyzed ions permitted, a minimum abundance setting of 2000 counts for the compound filter, minimum number of ions: 2, isotope model: common organic molecules with peak spacing tolerance 0.0025 *m*/*z*. The multivariate chemometric analysis were performed with Mass Profiler Professional (MPP) software version 12.61 (Agilent and Strand Life Sciences Pvt. Ltd., Santa Clara, CA, USA).

## 4. Conclusions

This study determined the behavior of maraviroc under stress conditions—neutral, acid and alkaline hydrolysis, oxidation and UVA irradiation—as well as traced its behavior under electrode oxidation processes. Nine products formed in these conditions were detected and identified using MS/MS fragmentation spectra. Two TPs were not previously described in the literature. The largest number of degradation products were formed under the irradiation of UVA. Among the seven photodegradation products, three of them were not formed under any other conditions. The chemometric analysis approach described in this work allowed the comprehensive study of the maraviroc forced degradation studies. The experiment showed that electrodes made of noble metals are more capable of imitating such reactions than the GC electrode. These findings indicate that electrode processes may successfully mimic oxidation reactions that are present in degradation processes and can therefore be considered as quick and relatively low-cost supplements to the commonly applied forced degradation procedures.

## Figures and Tables

**Table 1:** Accurate mass elemental composition and proposed structures of the analyzed compounds.

Name	Observed in Stress Condition	Retention Time [min]	Mass [*m*/*z*]		Mass Error [ppm]	Molecular Formula [M+H]^+^	Fragmentation MS/MS	
			Measured	Theoretical			Mass [*m*/*z*]	Ion Formula [M+H]^+^
Maraviroc		4.25	514.3349	514.3352	0.58	C_29_H_42_F_2_N_5_O	389.2397 280.1512 260.1438 235.1878 226.1587 205.1428 185.1227 * 144.0816 124.1026 117.0703 106.0656 99.0604 91.0540 79.0554	C_23_H_31_F_2_N_2_O C_16_H_20_F_2_NO C_16_H_19_FNO C_13_H_23_N_4_ C_16_H_20_N C_11_H_17_N_4_ C_22_H_35_N_5_* C_7_H_11_FNO C_8_H_14_N C_9_H_9_ C_7_H_8_N C_6_H_8_F C_7_H_7_ C_6_H_7_
TP1	UVA, Pt, GC	0.7	235.1921	235.1917	1.70	C_13_H_23_N_4_	207.1849 126.1023 110.0971 93.0697 82.0651 67.0551	C_13_H_23_N_2_ C_6_H_12_N_3_ C_7_H_12_N C_7_H_9_ C_5_H_8_N C_5_H_7_
TP2	NaOH	1.04	368.2793	368.2809	4.34	C_22_H_34_N_5_	243.1893 124.1122 96.0823 70.0672	C_16_H_23_N_2_ C_8_H_14_N C_6_H_10_N C_4_H_8_N
TP3	UVA	3.54	530.3306	530.3301	0.94	C_29_H_42_F_2_N_5_O_2_	405.2332 296.1450 247.1923 242.1535 205.1470 164.0888 144.0816 133.0620 124.1105 99.0612	C_23_H_31_F_2_N_2_O_2_ C_16_H_20_F_2_NO_2_ C_14_H_23_N_4_ C_16_H_20_NO C_11_H_17_N_4_ C_7_H_12_F_2_NO C_7_H_11_FNO C_9_H_9_O C_8_H_14_N C_6_H_8_F
TP4	UVA	3.71	530.3306	530.3301	0.94	C_29_H_42_F_2_N_5_O_2_	405.2352 296.1451 247.1933 205.1456 144.0830 133.0641 124.1134 122.0589 119.0672 110.0971 99.0631	C_23_H_31_F_2_N_2_O_2_ C_16_H_20_F_2_NO_2_ C_14_H_23_N_4_ C_11_H_17_N_4_ C_7_H_11_FNO C_9_H_9_O C_8_H_14_N C_7_H_8_NO C_6_H_9_F_2_ C_7_H_12_N C_6_H_8_F
TP5	UVA	4.12	530.3306	530.3301	0.94	C_29_H_42_F_2_N_5_O_2_	405.2355 296.1468 247.1897 242.1514 144.0831 133.0638 124.1123 122.0962 119.0652 110.0971 99.0653	C_23_H_31_F_2_N_2_O_2_ C_16_H_20_F_2_NO_2_ C_14_H_23_N_4_ C_16_H_20_NO C_7_H_11_FNO C_9_H_9_O C_8_H_14_N C_8_H_12_N C_6_H_9_F_2_ C_7_H_12_N C_6_H_8_F
TP6	UVA, H_2_O_2_, Au, Pt	4.25	530.3285	530.3301	3.02	C_29_H_42_F_2_N_5_O_2_	389.2387 280.1512 251.1860 144.0851 126.0948 117.0693 106.0652 99.0629 91.0536	C_23_H_31_F_2_N_2_O C_16_H_20_F_2_NO C_13_H_22_N_4_O C_7_H_11_FNO C_7_H_11_NO C_9_H_9_ C_7_H_8_N C_6_H_8_F C_7_H_7_
TP7	UVA, H_2_O_2_, Au, Pt	4.43	530.3312	530.3301	2.07	C_29_H_42_F_2_N_5_O_2_	405.2344 280.1508 251.1867 184.1311 * 144.0810 126.0933 117.0701 106.0656 99.0656 81.0707 67.0536	C_23_H_31_F_2_N_2_O_2_ C_16_H_20_F_2_NO C_13_H_22_N_4_O C_22_H_32_N_4_O* C_7_H_11_FNO C_7_H_12_NO C_9_H_9_ C_7_H_8_N C_6_H_8_F C_6_H_9_ C_5_H_7_
TP8	UVA	4.49	546.3284	546.3250	6.22	C_29_H_42_F_2_N_5_O_3_	514.2988 389.2441 280.1550 243.1861 185.1193 * 144.0821 140.0940 * 124.1092 119.0640	C_29_H_42_F_2_N_5_O C_23_H_31_F_2_N_2_O C_16_H_20_F_2_NO C_16_H_23_N_2_ C_22_H_34_N_4_O * C_7_H_11_FNO C_14_H_24_N_4_O_2_* C_8_H_14_N C_6_H_9_F_2_
TP9	GC, Au	5.7	528.3137	528.3145	1.51	C_29_H_40_F_2_N_5_O_2_	294.1297 252.1195 240.1364 148.0765 131.0492 126.1029 119.0666 110.0966 106.0653 99.0611 93.0703 79.0543 67.0554	C_16_H_18_F_2_NO_2_ C_14_H_16_F_2_NO C_16_H_18_NO C_9_H_10_O C_9_H_7_O C_6_H_12_N_3_ C_6_H_9_F_2_ C_7_H_12_N C_7_H_8_N C_6_H_8_F C_7_H_9_ C_6_H_7_ C_5_H_7_

* Double-charged ion.

**Table 2:** Stress conditions applied to maraviroc degradation.

Stress Conditions	Diluting Solvent	Exposure Conditions	Duration (h)
Acid hydrolysis	0.2 M HCl	80 °C	3
Alkaline hydrolysis	0.2 M NaOH	80 °C	3
Neutral hydrolysis	H_2_O	80 °C	3
Oxidation	1% H_2_O_2_	Room temperature	3
Photolysis (UVA)	H_2_O	Room temperature	1

**Table 3:** LC–MS parameters.

Device	Parameter	Value
LC	Solvents	A—0.1% aqueous solution of HCOOH B—acetonitrile
	Gradient	10% B to 55% B
	Analysis time	8 min
	Post-time equilibration	2 min
	Flow rate	0.3 mL min^−1^
	Injection volume	1.5 µL
	Column temperature	40 °C
MS	Ion source	Electrospray (ESI)
	Mode	Positive
	Source temperature	325 °C
	Drying gas flow	10 L min^−1^
	Nebulizer pressure	40 psig
	Capillary voltage	3500 V
	Fragmentor voltage	150 V
	Skimmer voltage	65 V
	Octopole voltage	750 V
	Mass range	60–1050 *m*/*z*
	Acquisition rate	2 spectra s^−1^ *

* In the MS/MS mode and 1.5 spectra s^−1^ in the TOF mode.

## Data Availability

Data are contained within this article or the Supplementary Materials.

## Supplementary Materials

The following supporting information can be downloaded at: https://www.mdpi.com/article/10.3390/molecules28031195/s1, Figure S1: MS/MS spectrum and fragmentation pattern of single-charged maraviroc; Figure S2: MS/MS spectrum and fragmentation pattern of double-charged maraviroc; Figure S3: MS/MS spectrum and fragmentation pattern of TP1 metabolite; Figure S4: MS/MS spectrum and fragmentation pattern of TP2; Figure S5: MS/MS spectrum and fragmentation pattern of single-charged TP3; Figure S6: MS/MS spectrum and fragmentation pattern of double-charged TP3; Figure S7: MS/MS spectrum and fragmentation pattern of single-charged TP4; Figure S8: MS/MS spectrum and fragmentation pattern of double-charged TP4; Figure S9: MS/MS spectrum and fragmentation pattern of single-charged TP5; Figure S10: MS/MS spectrum and fragmentation pattern of double-charged TP5; Figure S11: MS/MS spectrum and fragmentation pattern of single-charged TP6; Figure S12: MS/MS spectrum and fragmentation pattern of double-charged TP6; Figure S13: MS/MS spectrum and fragmentation pattern of single-charged TP7; Figure S14: MS/MS spectrum and fragmentation pattern of double-charged TP7; Figure S15: MS/MS spectrum and fragmentation pattern of single-charged TP8; Figure S16: MS/MS spectrum and fragmentation pattern of double-charged TP8; Figure S17: MS/MS spectrum and fragmentation pattern of TP9.

## Sample Availability

Samples of the compounds are not available.

## References

[1] Skibiński R., Trawiński J. (2017). Application of an Untargeted Chemometric Strategy in the Impurity Profiling of Pharmaceuticals: An Example of Amisulpride. J. Chromatogr. Sci..

[2] Melo S.R.D.O., Homem-de-Mello M., Silveira D., Simeoni L.A. (2014). Advice on Degradation Products in Pharmaceuticals: A Toxicological Evaluation. PDA J. Pharm. Sci. Technol..

[3] Holm R., Elder D.P. (2016). Analytical Advances in Pharmaceutical Impurity Profiling. Eur. J. Pharm. Sci..

[4] Gómez-Canela C., Bolivar-Subirats G., Tauler R., Lacorte S. (2017). Powerful Combination of Analytical and Chemometric Methods for the Photodegradation of 5-Fluorouracil. J. Pharm. Biomed. Anal..

[5] De Luca M., Ioele G., Grande F., Platikanov S., Tauler R., Ragno G. (2020). Photostability Study of Multicomponent Drug Formulations via MCR-ALS: The Case of the Hydrochlorothiazide-Amiloride Mixture. J. Pharm. Biomed. Anal..

[6] Luca M.D., Ragno G., Ioele G., Tauler R. (2014). Multivariate Curve Resolution of Incomplete Fused Multiset Data from Chromatographic and Spectrophotometric Analyses for Drug Photostability Studies. Anal. Chim. Acta.

[7] Skibiński R., Trawiński J., Komsta Ł., Murzec D. (2018). Characterization of Forced Degradation Products of Toloxatone by LC-ESI-MS/MS. Saudi Pharm. J..

[8] Cordella B.Y.C., Krull I.S. (2012). PCA: The Basic Building Block of Chemometrics. Analytical Chemistry.

[9] Roberto de Alvarenga Junior B., Lajarim Carneiro R. (2019). Chemometrics Approaches in Forced Degradation Studies of Pharmaceutical Drugs. Molecules.

[10] Torres S., Brown R., Szucs R., Hawkins J.M., Zelesky T., Scrivens G., Pettman A., Taylor M.R. (2015). The Application of Electrochemistry to Pharmaceutical Stability Testing—Comparison with in Silico Prediction and Chemical Forced Degradation Approaches. J. Pharm. Biomed. Anal..

[11] Singh S., Wang J., Cinti S. (2022). Review—An Overview on Recent Progress in Screen-Printed Electroanalytical (Bio)Sensors. ECS Sens. Plus.

[12] García-Miranda Ferrari A., Rowley-Neale S.J., Banks C.E. (2021). Screen-Printed Electrodes: Transitioning the Laboratory in-to-the Field. Talanta Open.

[13] Veljkovic N., Vucicevic J., Tassini S., Glisic S., Veljkovic V., Radi M. (2015). Preclinical Discovery and Development of Maraviroc for the Treatment of HIV. Expert Opin. Drug Discov..

[14] Chakravarthy V.K., Sankar D. (2012). Gowri Stability Indicating UHPLC Method for Determination of Maraviroc and Its Degradents/Impurities in Bulk and Pharmaceutical Formulation. Rasayan J. Chem..

[15] Sait S., Lalitha G., Geetha M. (2013). Stability Indicating UHPLC Method for the Assay of Maraviroc in Bulk and in Formulations. Int. J. Drug Dev. Res..

[16] Chilukuri M., Hussainreddy K., Narayanareddy P., Venkataramana M. (2014). A Validated Stability-Indicating UPLC Method for the Determination of Impurities in Maraviroc. J. Chromatogr. Sci..

[17] Trawiński J., Wroński M., Skibiński R. (2022). Efficient Removal of Anti-HIV Drug-Maraviroc from Natural Water by Peroxymonosulfate and TiO_2_ Photocatalytic Oxidation: Kinetic Studies and Identification of Transformation Products. J. Environ. Manag..

