# Supplementary material for: Multivariate Chemometric Comparison of Forced Degradation and Electrochemical Oxidation LC–MS Profiles of Maraviroc
Source: Molecules. 2023 Jan 25;28(3):1195. doi: 10.3390/molecules28031195 (PMC9920961; doi:10.3390/molecules28031195)
Supplement: Supplementary file 1 [file molecules-28-01195-s001.zip › molecules-2146117-supplementary.pdf]

Supplementary Materials

Multivariate Chemometric Comparison of Forced Degradation and Electrochemical Oxidation LC–MS Profiles of Maraviroc

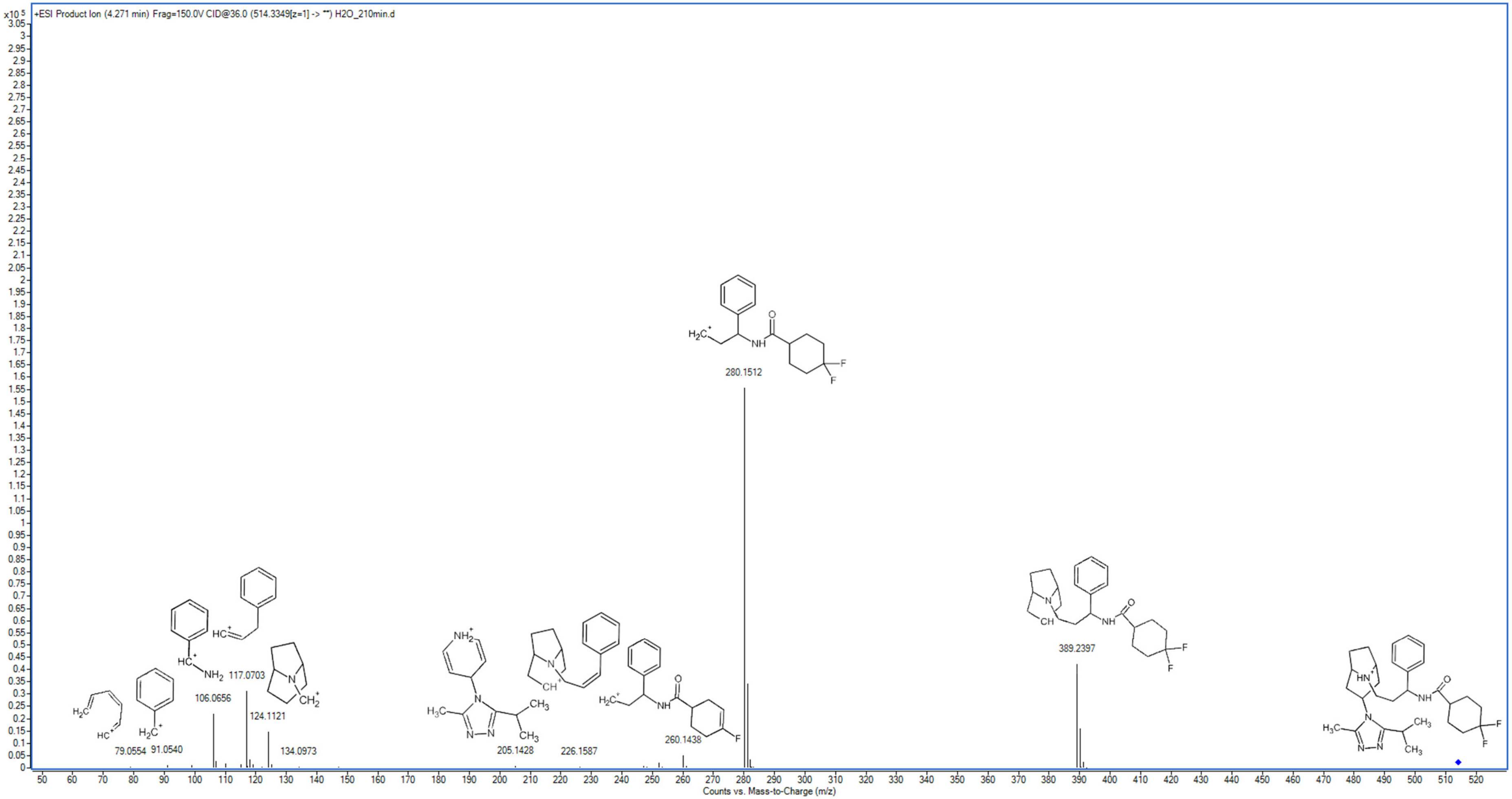

Figure S1. MS/MS spectrum and fragmentation pattern of single-charged maraviroc.

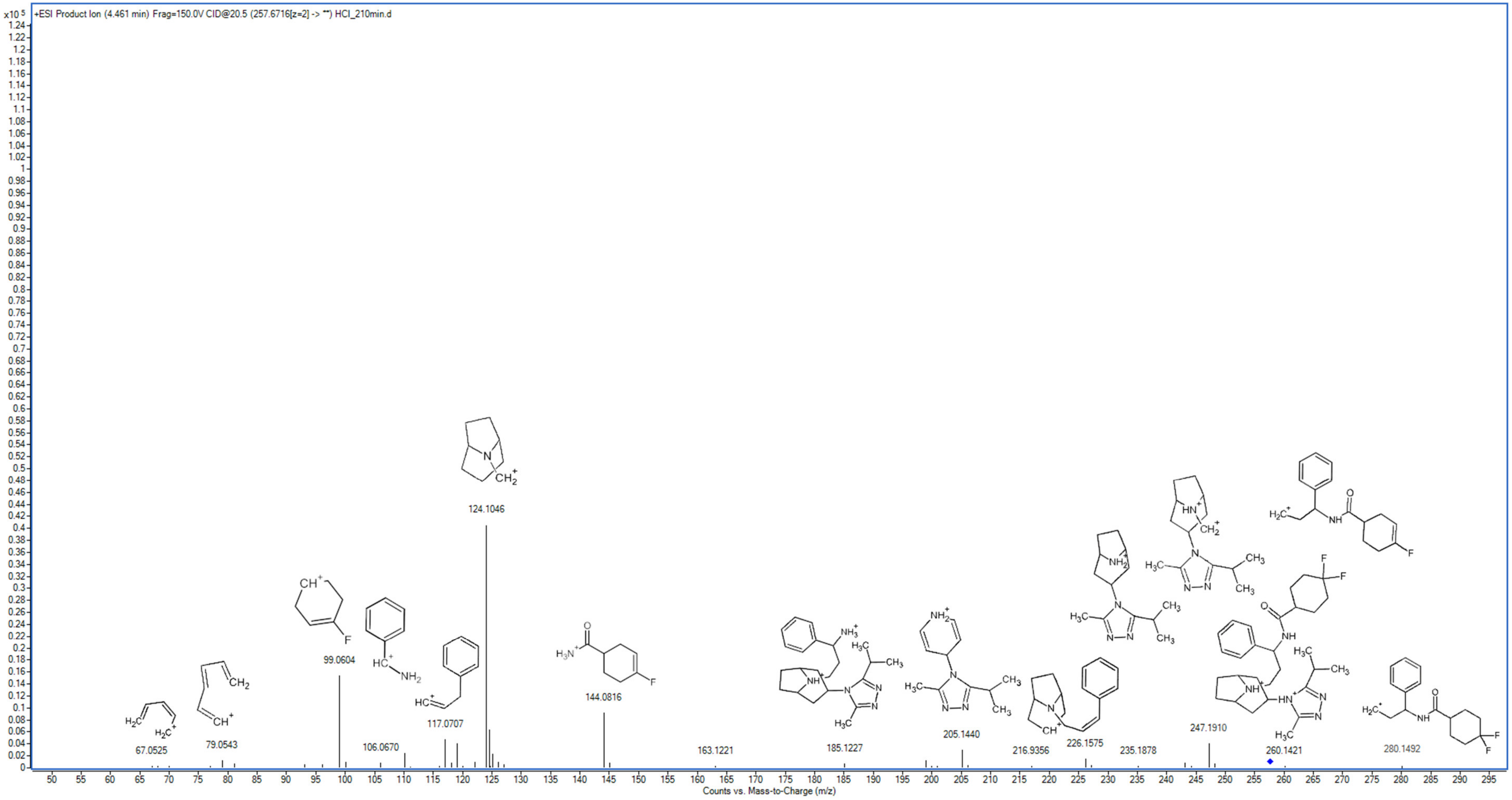

Figure S2. MS/MS spectrum and fragmentation pattern of double-charged maraviroc.

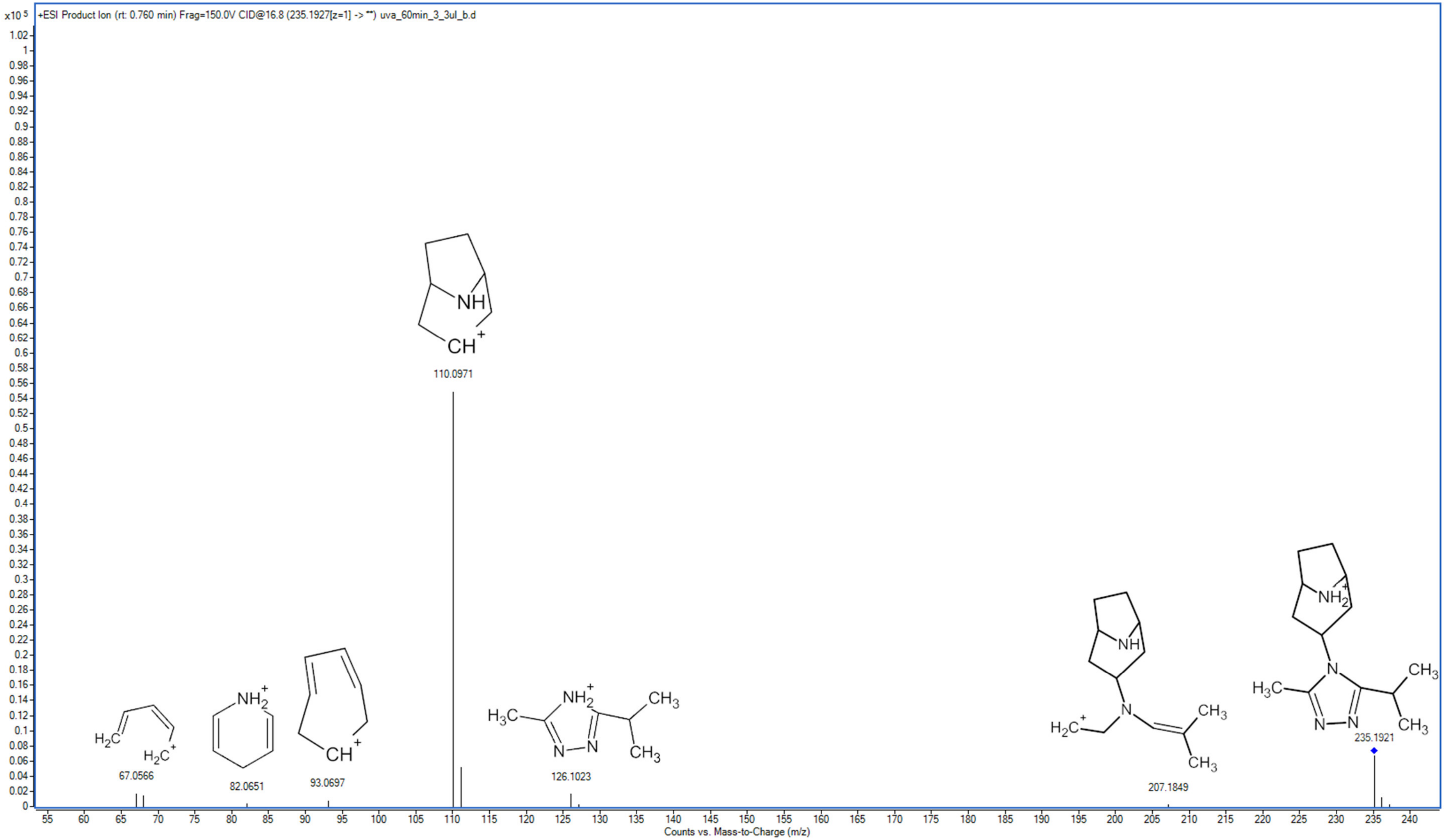

Figure S3. MS/MS spectrum and fragmentation pattern of TP1 metabolite.

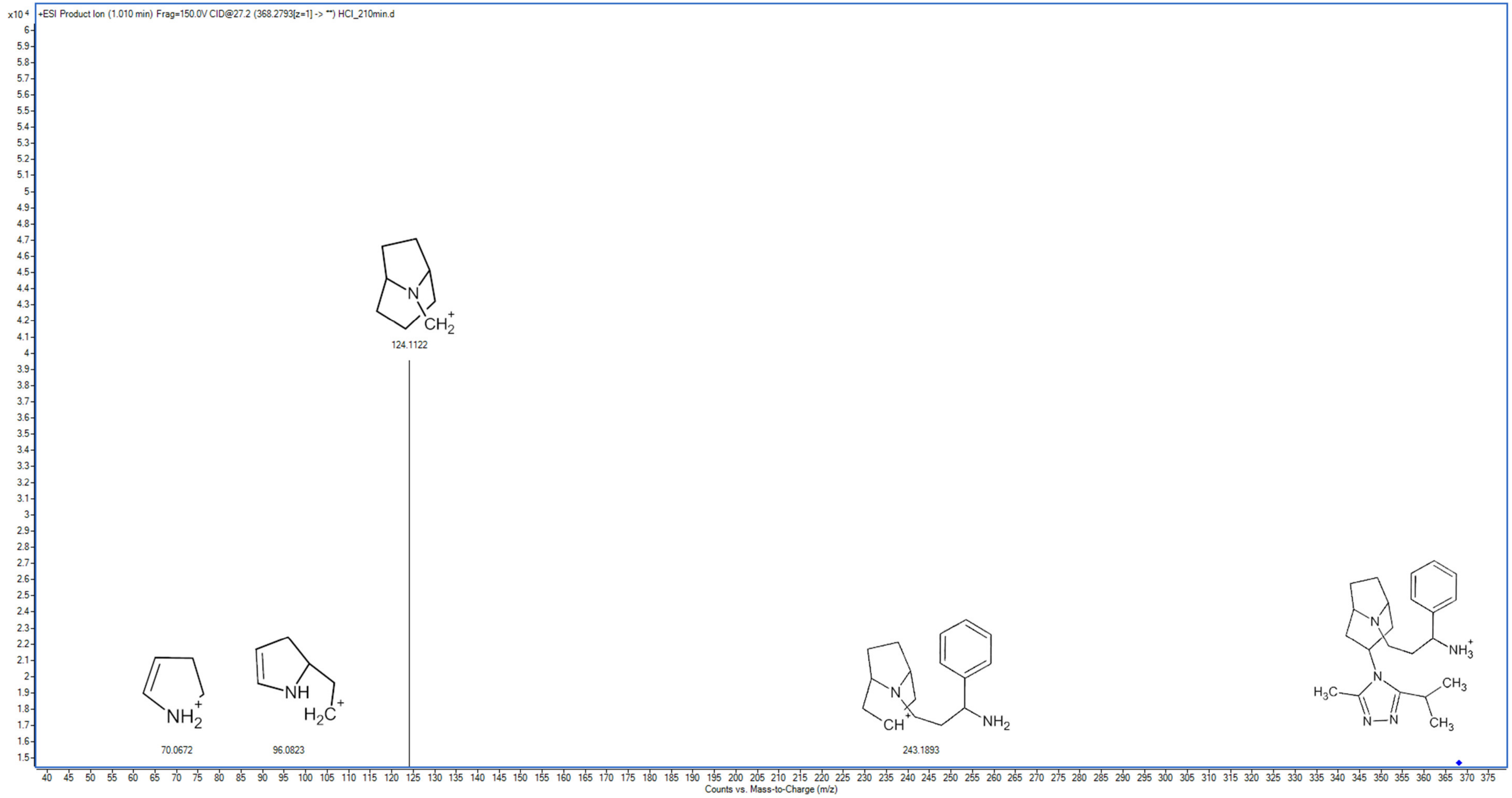

Figure S4. MS/MS spectrum and fragmentation pattern of TP2 metabolite.

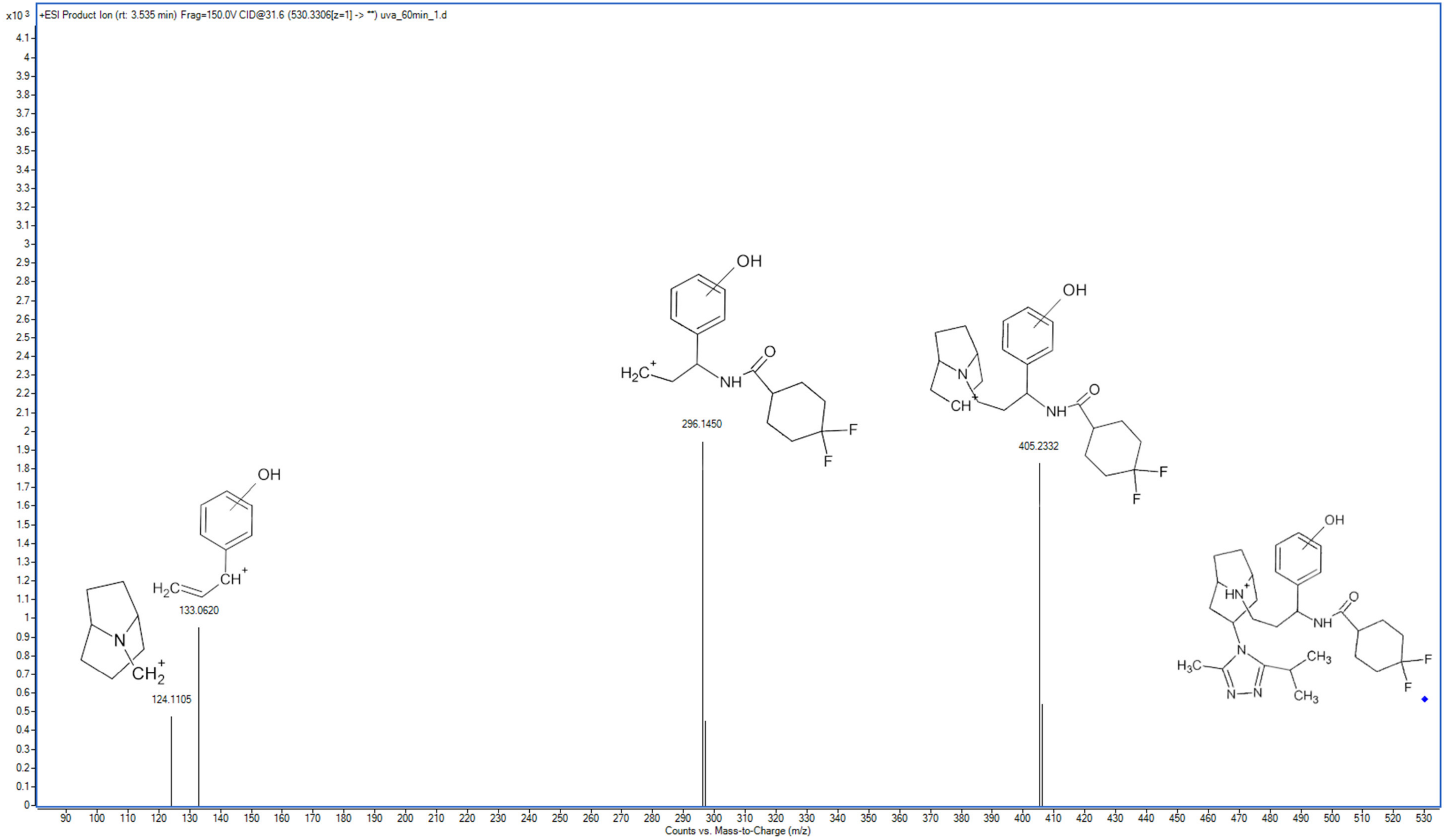

Figure S5. MS/MS spectrum and fragmentation pattern of single-charged TP3.

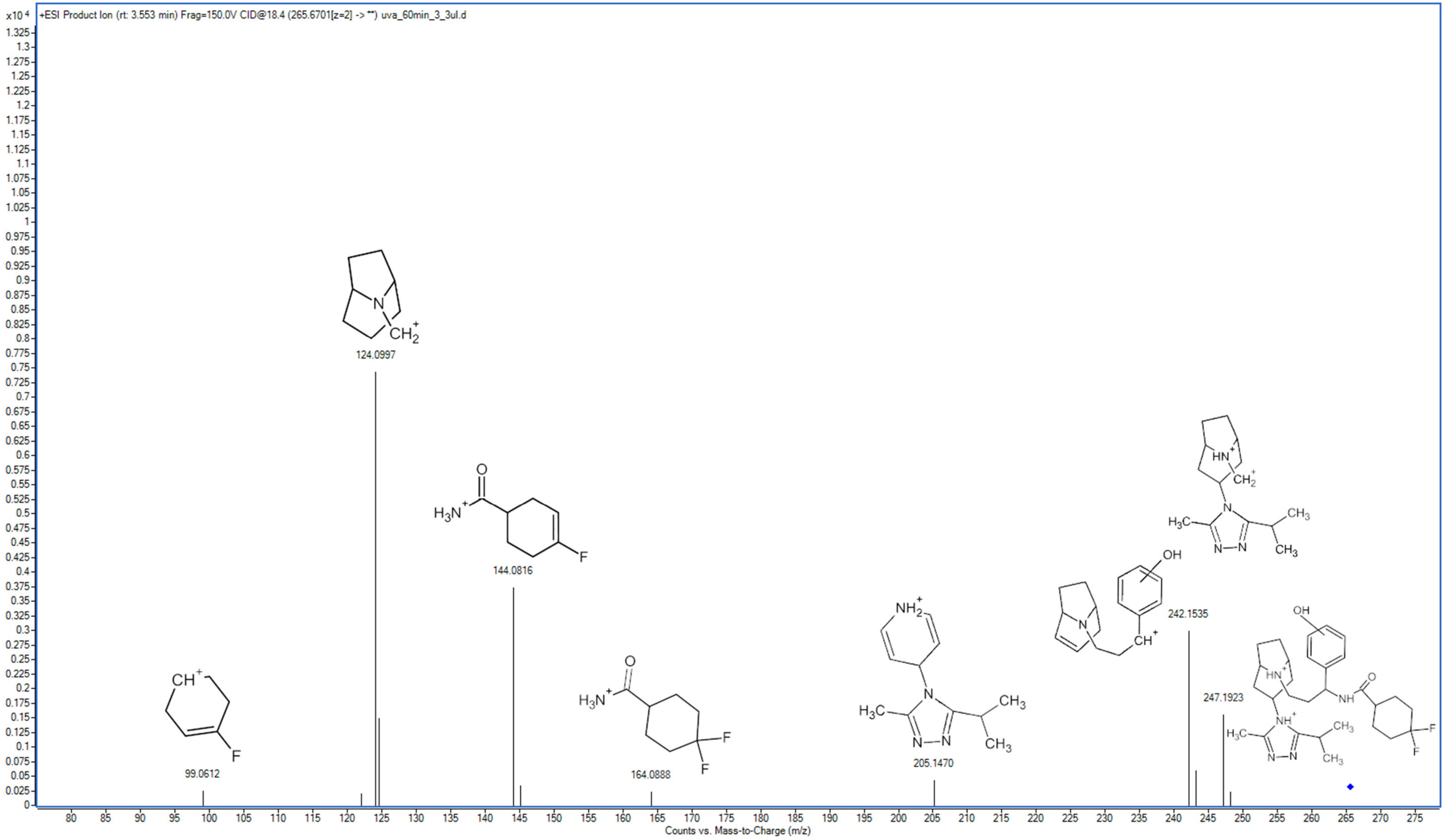

Figure S6. MS/MS spectrum and fragmentation pattern of double-charged TP3.

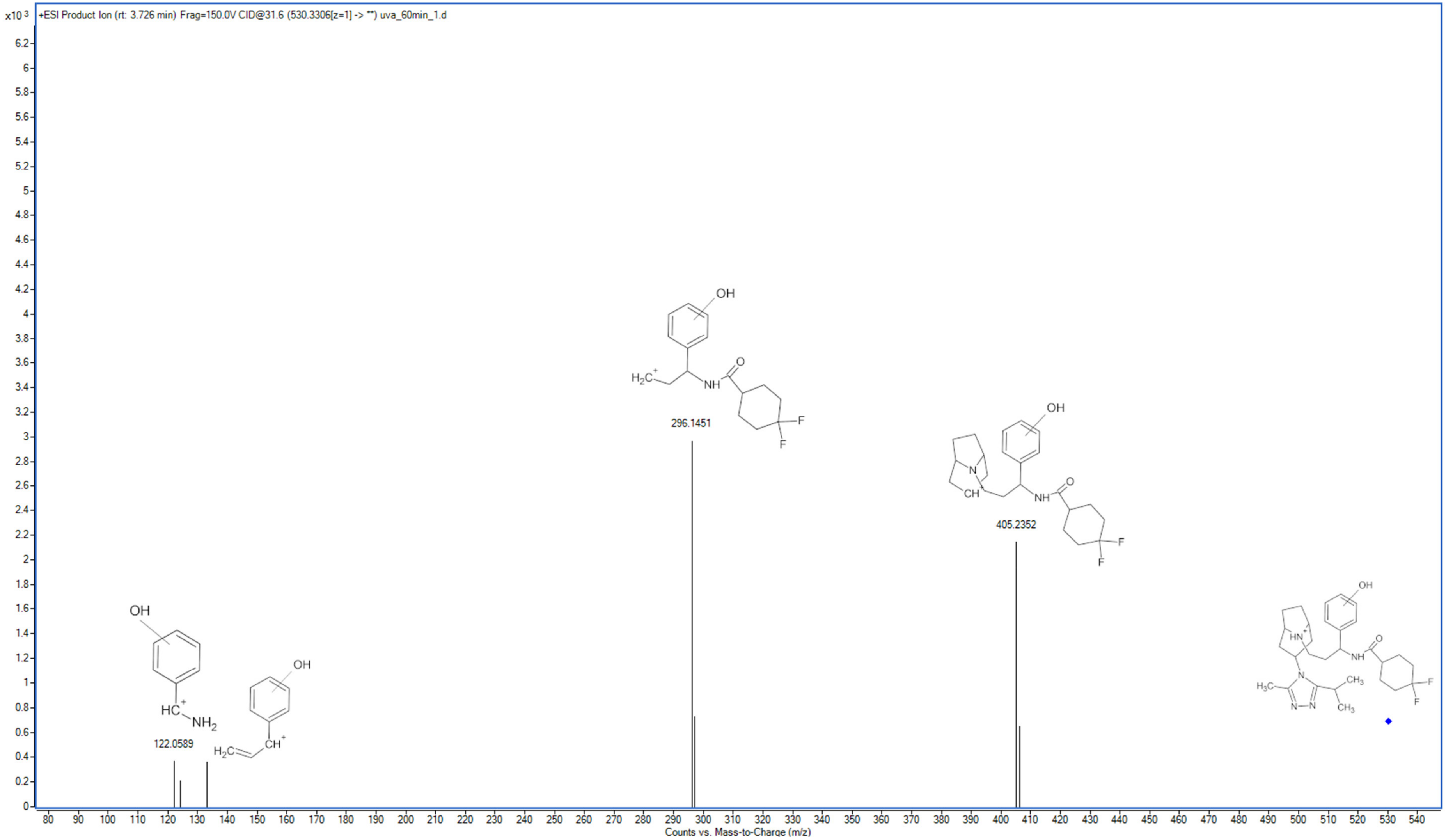

Figure S7. MS/MS spectrum and fragmentation pattern of single-charged TP4.

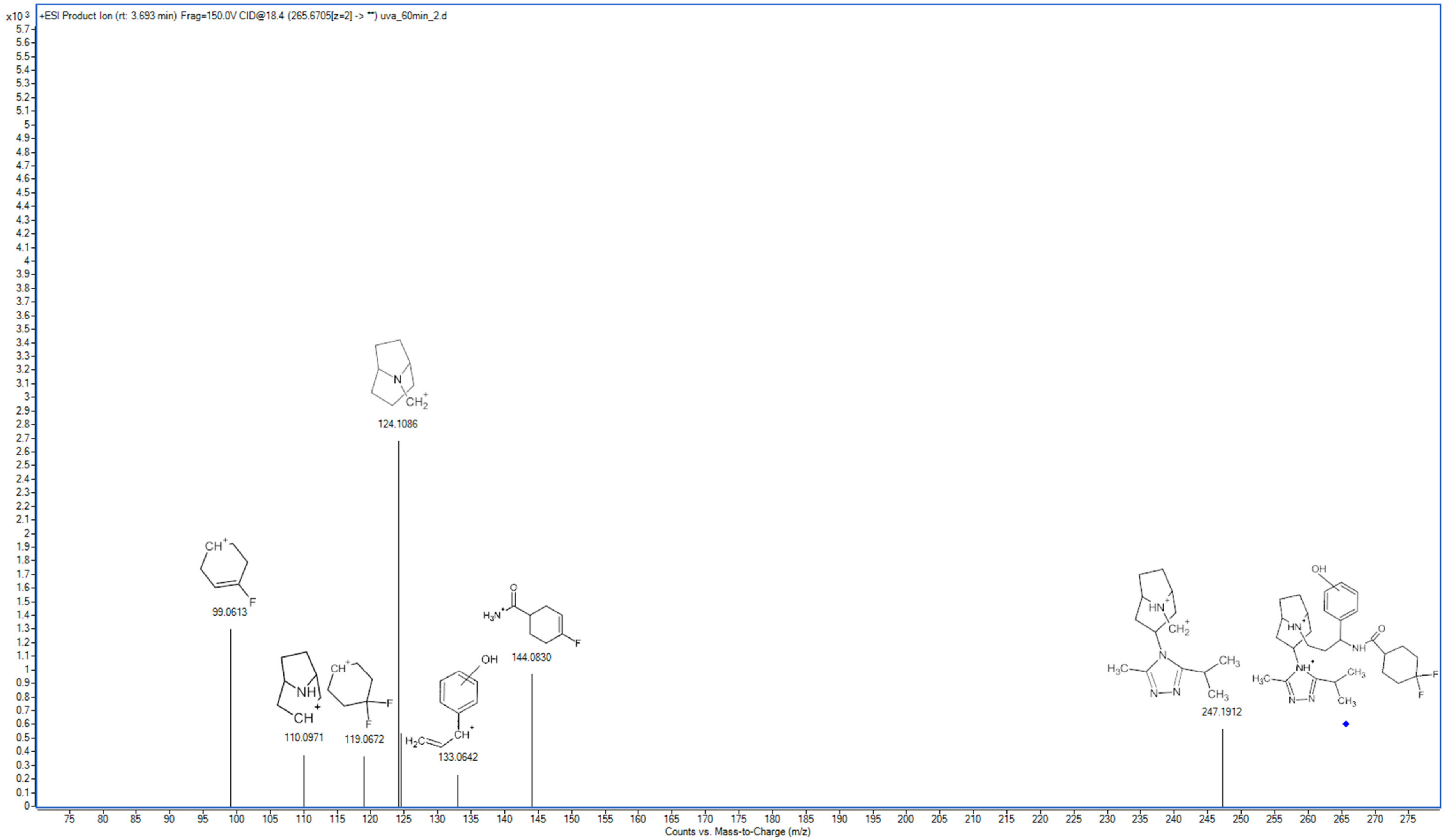

Figure S8. MS/MS spectrum and fragmentation pattern of double-charged TP4.

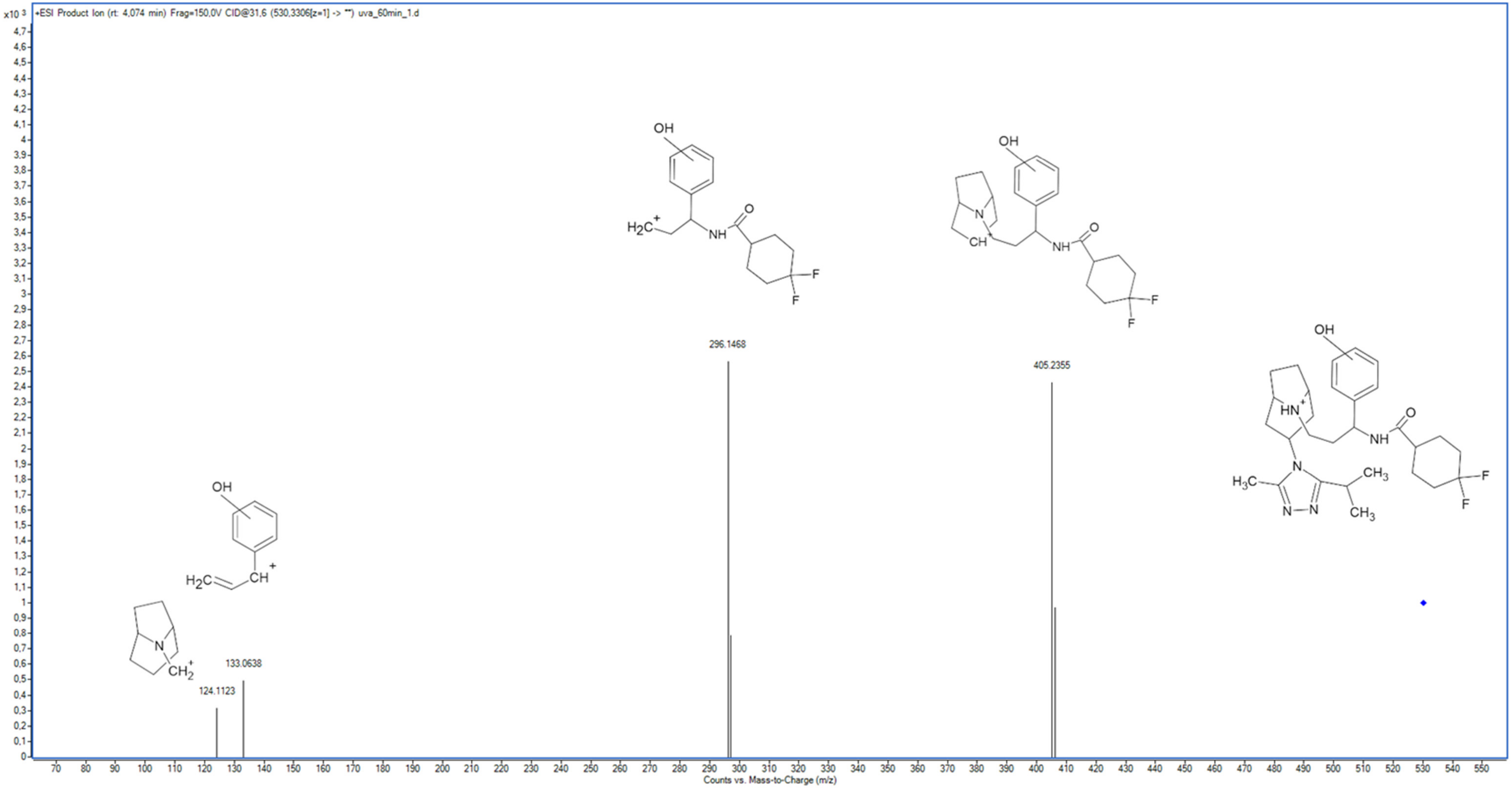

Figure S9. MS/MS spectrum and fragmentation pattern of single-charged TP5.

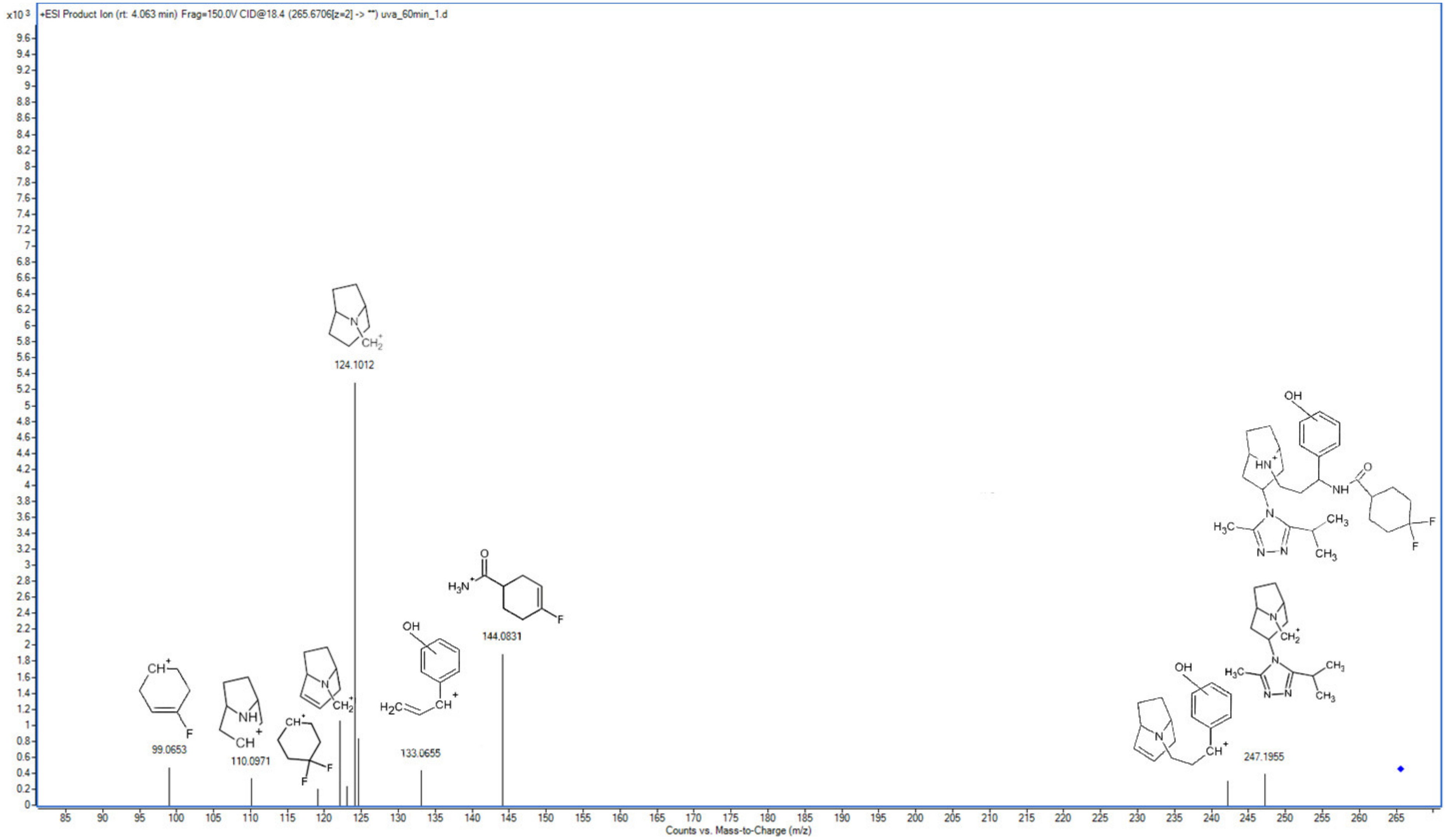

Figure S10. MS/MS spectrum and fragmentation pattern of double-charged TP5.

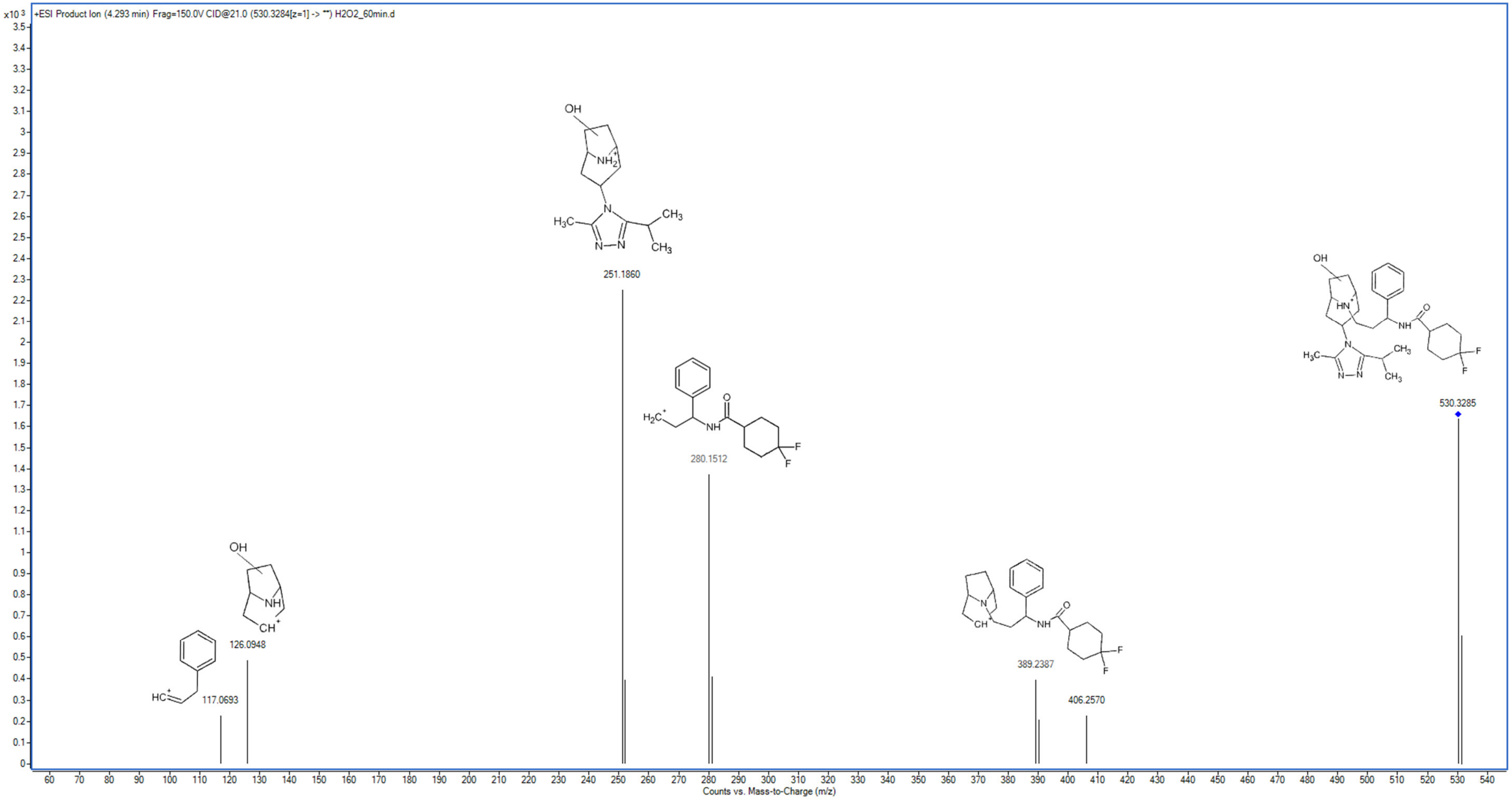

Figure S11. MS/MS spectrum and fragmentation pattern of single-charged TP6.

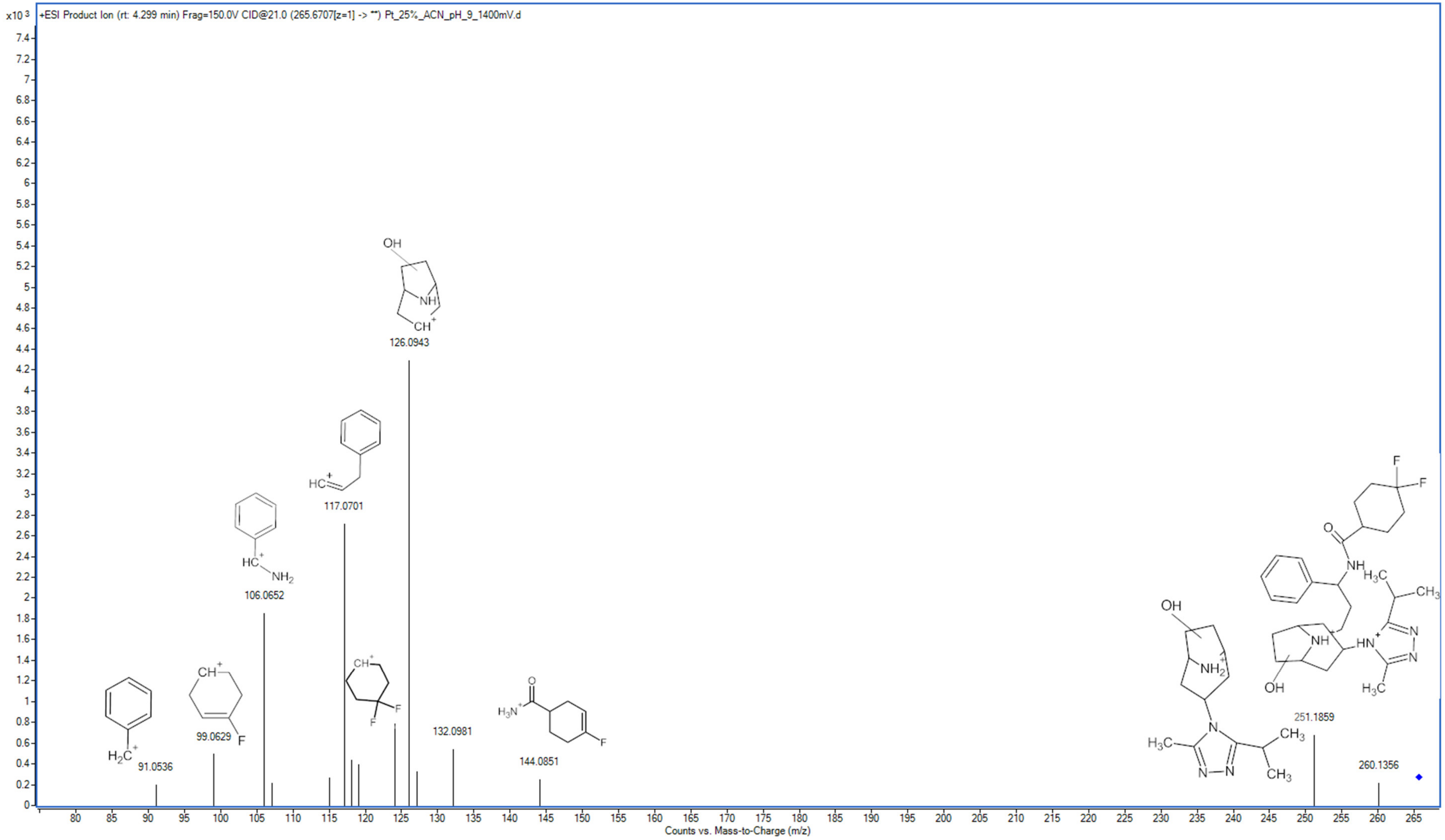

Figure S12. MS/MS spectrum and fragmentation pattern of double-charged TP6.

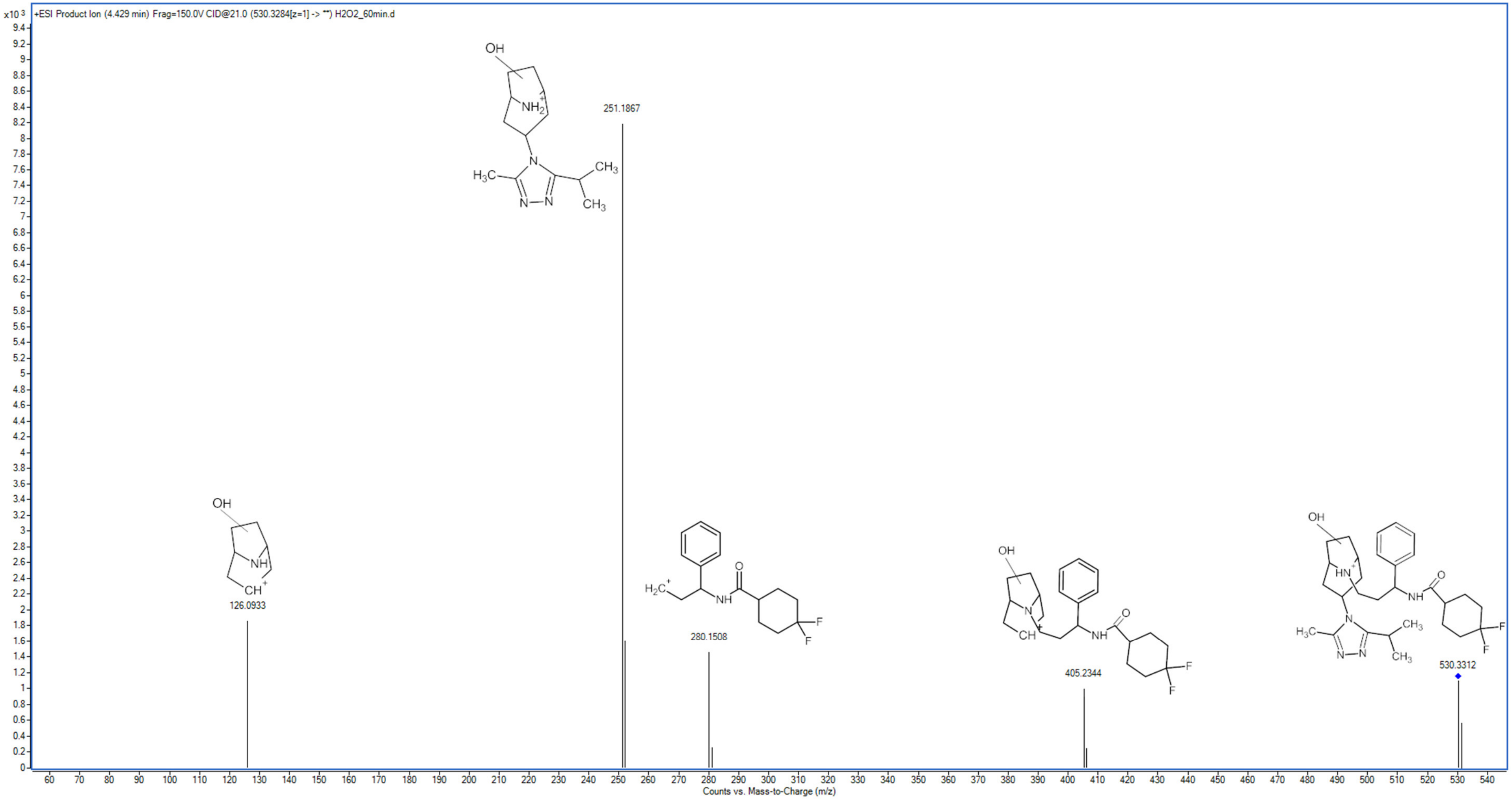

Figure S13. MS/MS spectrum and fragmentation pattern of single-charged TP7.

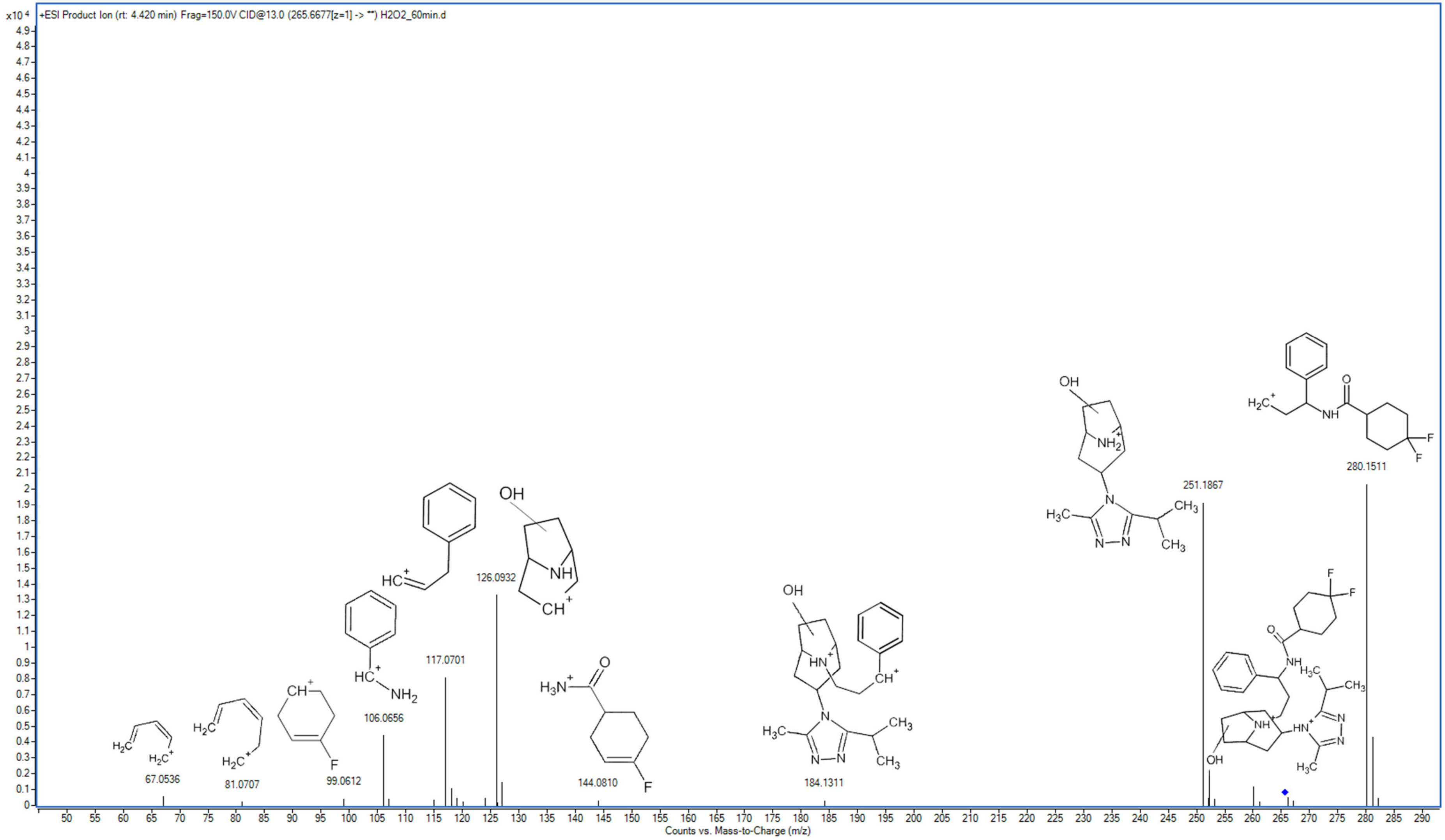

Figure S14. MS/MS spectrum and fragmentation pattern of double-charged TP7.

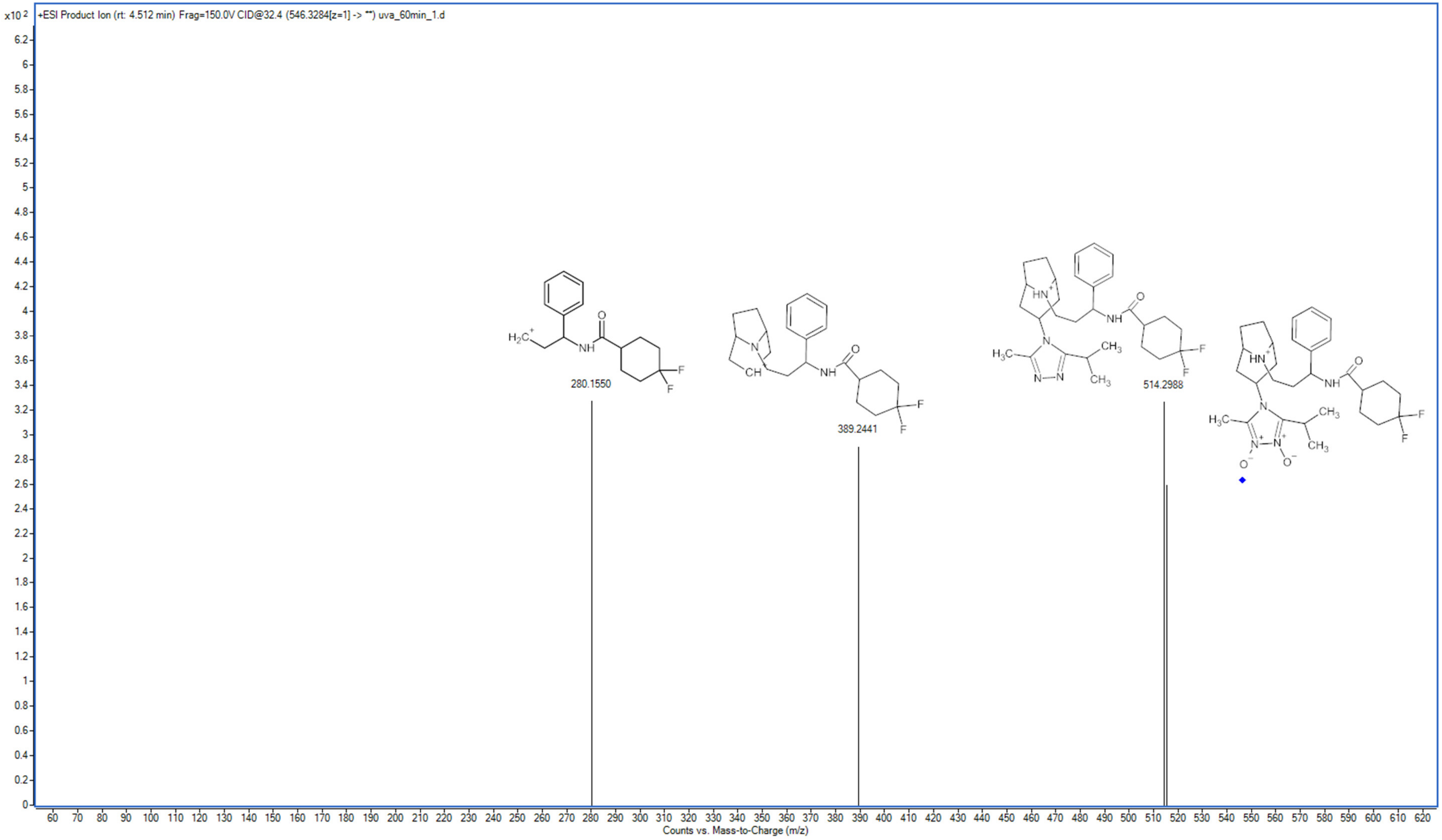

Figure S15. MS/MS spectrum and fragmentation pattern of single-charged TP8.

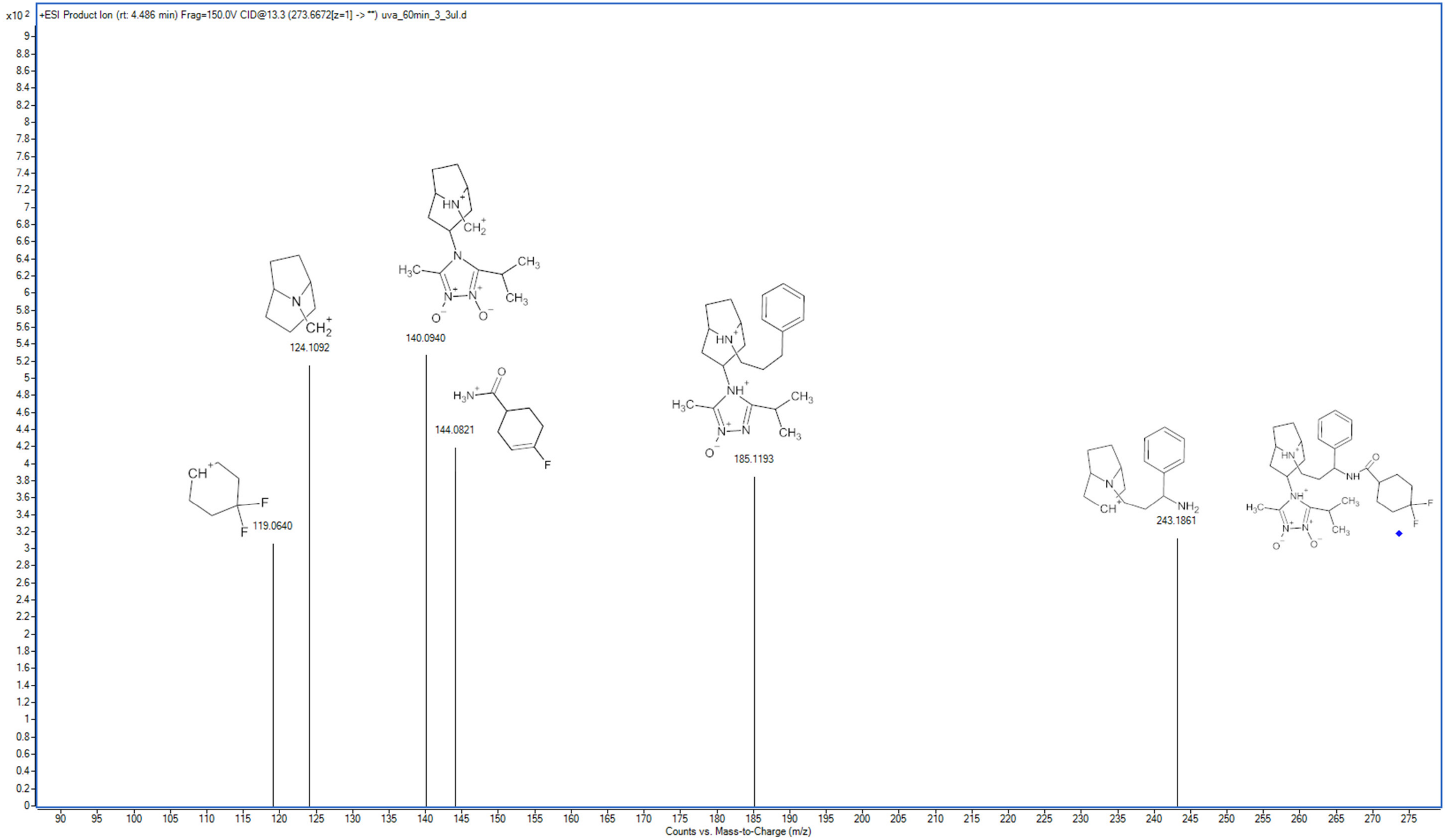

Figure S16. MS/MS spectrum and fragmentation pattern of double-charged TP8.

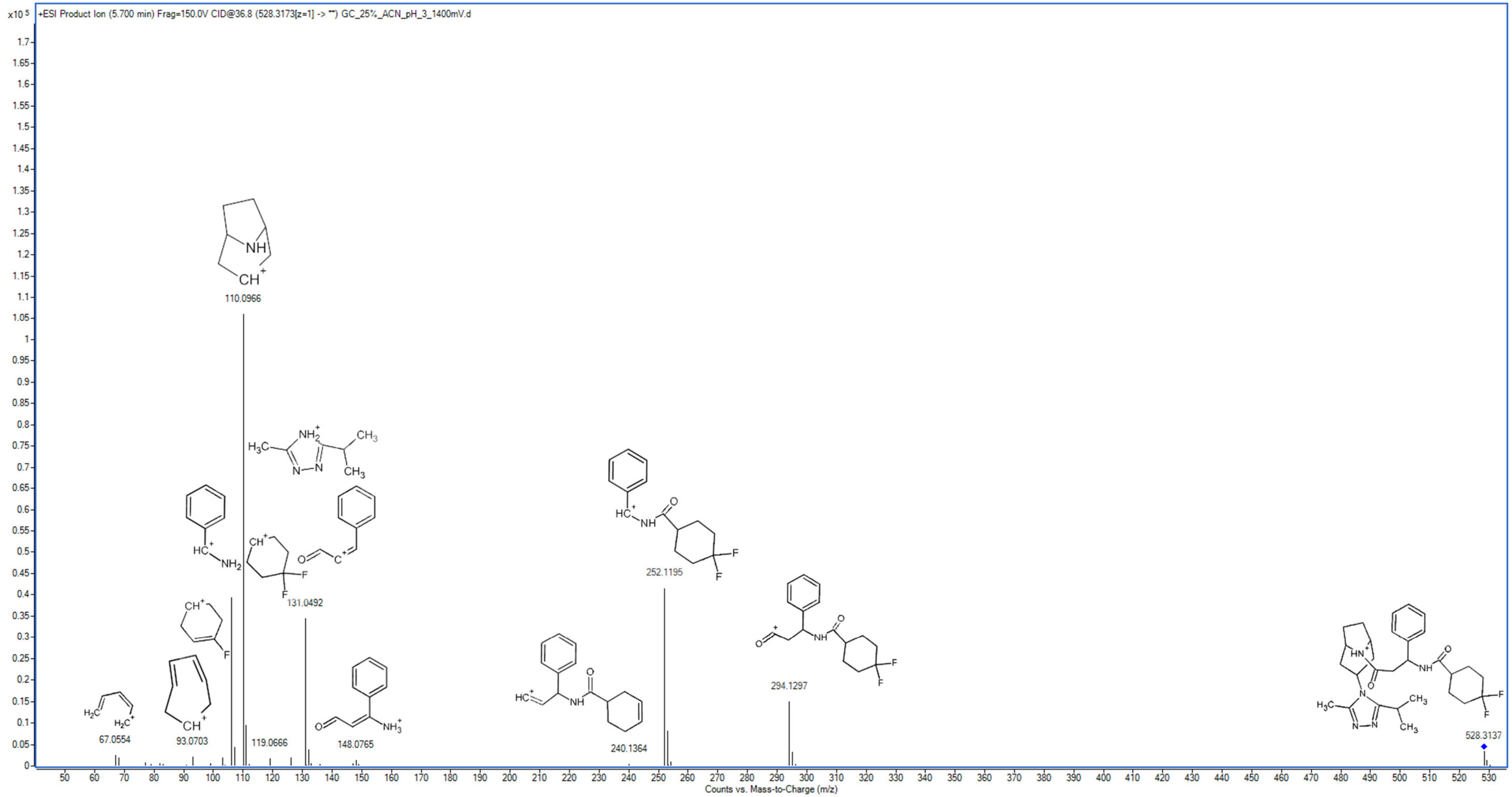

Figure S17. MS/MS spectrum and fragmentation pattern of TP9.
